# Diffusion tensor imaging-functional MRI fusion reveals disrupted white matter structure–function coupling in HIV-associated asymptomatic neurocognitive impairment

**DOI:** 10.3389/fnins.2026.1793111

**Published:** 2026-04-16

**Authors:** Wei Wang, Zhongkai Zhou, Ruichen Ren, Budong Chen, Wenru Gong, Chao Yuan, Tiantian Tian, Siyu Feng, Yanbin Shi, Hongjun Li, Lingling Zhao

**Affiliations:** 1 Department of Radiology, Beijing Youan Hospital, Capital Medical University, Beijing, China; 2 Medical Imaging Department of Henan Infectious Disease Hospital, Zhengzhou, China

**Keywords:** HIV-associated asymptomatic neurocognitive impairment, diffusion tensor imaging, resting-state fMRI, white matter structure–function coupling, SWALFF, dynamic SWALFF, white matter dys-coupling index

## Abstract

**Objective:**

Conventionally, blood oxygen level-dependent (BOLD) signals derived from resting-state functional magnetic resonance imaging (rs-fMRI) are attributed to gray matter, but recent evidence confirms stable low-frequency oscillations within white matter. While structure–function coupling is pivotal in neuropsychiatry, it remains underexplored in HIV-associated neurocognitive disorders (HAND). Focusing on Asymptomatic Neurocognitive Impairment (ANI), the earliest stage of HAND, this study establishes a white matter skeleton-based fusion framework integrating diffusion tensor imaging (DTI) and rs-fMRI to investigate underlying mechanisms.

**Methods:**

We enrolled 47 patients with ANI and 48 matched healthy controls. Fractional anisotropy (FA) images from DTI and BOLD signals derived from rs-fMRI were projected onto a unified white matter skeleton to achieve structure–function spatial alignment. FA, skeleton-based white matter amplitude of low-frequency fluctuations (SWALFF), and its dynamic variability (dSWALFF) were calculated. Group differences in white matter structure and function were assessed, with structure–function coupling examined in regions showing overlapping FA-SWALFF and FA-dSWALFF alterations. Additionally, a novel White Matter Dys-coupling Index (WDI) was proposed to quantify the deviation between structural integrity and functional activity and evaluate its clinical relevance.

**Results:**

Compared to controls, ANI patients exhibited widespread FA reductions and increased mean diffusivity (MD) and radial diffusivity (RD), indicating diffuse demyelination. Functionally, a spatial dissociation emerged: SWALFF was reduced in posterior occipital pathways (left vertical occipital fasciculus, forceps major), whereas SWALFF and dSWALFF were elevated in prefrontal pathways (forceps minor). Overlapping regions revealed complex coupling patterns, ranging from concordant decline to compensatory upregulation and decoupling. The interaction between FA and dSWALFF further highlighted instability in dynamic regulation. The WDI was significantly correlated with infection duration, immune status, and cognitive domain scores.

**Conclusion:**

This study identifies a characteristic “coupling imbalance” in the white matter of ANI patients, defined by the coexistence of structural degeneration and functional reorganization. We propose the WDI as a quantitative metric for this deviation. Its significant associations with clinical and cognitive metrics suggest its potential as a neuroimaging biomarker for the early identification and mechanistic understanding of HAND.

## 1 Introduction

Although combination antiretroviral therapy (cART) has markedly prolonged survival in people living with HIV and effectively suppressed peripheral viral load (Heaton et al., 2010), its protective effects on the central nervous system (CNS) remain limited and may not fully prevent the persistent impact of HIV-associated neurotoxicity (Simioni et al., 2010). Evidence indicates that neurocognitive impairment remains common even among individuals with long-term viral suppression (Sacktor et al., 2016). HIV-associated neurocognitive disorders (HAND) comprise a spectrum of neuropsychiatric syndromes directly or indirectly attributable to HIV infection, characterized primarily by deficits in attention, working memory, processing speed, and fine motor coordination (Winston and Spudich, 2020). These impairments can substantially compromise quality of life, occupational functioning, and treatment adherence, thereby imposing a considerable burden on families and society (Woods et al., 2017). According to the 2007 Frascati criteria, HAND is classified into three subtypes: asymptomatic neurocognitive impairment (ANI), mild neurocognitive disorder (MND), and HIV-associated dementia (HAD) (Antinori et al., 2007).

In the era of cART, the clinical spectrum of HAND has shifted from severe impairment dominated by HAD toward milder phenotypes, with ANI now emerging as the most prevalent subtype (Heaton et al., 2010). Although individuals with ANI typically report no overt subjective symptoms or functional impairment, studies suggest that cognitive decline can persist and may affect higher-order everyday functions, such as driving, employment, and medication adherence (Clifford and Ances, 2013; Gouse et al., 2021). Importantly, individuals with ANI have a 2-6-fold higher risk of progressing to symptomatic cognitive impairment than cognitively normal individuals (Grant et al., 2014). As an early stage in the HAND continuum, ANI may be partially reversible and is therefore considered a potential “window of opportunity” for intervention (Ellis et al., 2007). At present, HAND diagnosis largely relies on standardized neuropsychological testing across multiple cognitive domains, a process that is time-consuming and operationally complex and is subject to false-positive findings, thereby limiting effective identification before neuropsychiatric symptoms become apparent (Clifford and Ances, 2013; Kamminga et al., 2017). Therefore, there is an urgent clinical and research need to develop sensitive and objective tools for early detection, particularly for identifying impairment at the ANI stage.

In recent years, advances in neuroimaging have provided important opportunities for the objective assessment and mechanistic investigation of HAND. Resting-state functional magnetic resonance imaging (rs-fMRI) studies indicate that HIV infection is associated with disrupted functional connectivity in prefrontal-parietal/occipital and cortico-striatal pathways, alongside reduced within- and between-network connectivity and aberrant topological integration and segregation across large-scale systems, including the default mode network (DMN), executive control network (CON), and salience network (SAL) (Thomas et al., 2013; Ortega et al., 2015; Ann et al., 2016; Li et al., 2019). Diffusion tensor imaging (DTI) studies further suggest that early HIV infection may already involve decreased fractional anisotropy (FA) and increased mean diffusivity (MD) and radial diffusivity (RD) in key white-matter regions such as the corpus callosum and corona radiata, even during clinically asymptomatic stages; with disease progression, these abnormalities appear to extend to multiple regions, including the frontal, parietal, and temporal lobes (Cao et al., 2015; Ragin et al., 2015; O'Connor et al., 2017). However, although these MRI modalities characterize HIV-related brain injury from complementary perspectives, functional and structural alterations are unlikely to be independent; instead, they may reflect multilevel structure–function coupling (Rykhlevskaia et al., 2008; Liu et al., 2022). Notably, the characteristics of this coupling in the context of HIV infection have yet to be systematically characterized or comprehensively understood.

Traditionally, the blood oxygen level-dependent (BOLD) signal measured with rs-fMRI has been considered to originate primarily from gray matter (GM) (Biswal et al., 1995). However, accumulating evidence indicates that white matter is not a functionally “silent” region; rather, it exhibits stable and reproducible functional activity that cannot be attributed to random noise (Zuo et al., 2010; Ji et al., 2017; Li et al., 2020). Recent studies have further demonstrated that white matter functional signals reflect meaningful metabolic and network-level activity, as revealed by combined fluorodeoxyglucose positron emission tomography-fMRI (fPET-fMRI) and individual white-matter functional connectome analyses (Zuo et al., 2010; Li et al., 2020; Li J. et al., 2023). Importantly, the topological organization of these white-matter functional networks closely parallels the fiber-tract architecture revealed by DTI (Peer et al., 2017; Gore et al., 2019) and has shown sensitivity to pathological states in disorders such as Parkinson’s disease, Alzheimer’s disease, and schizophrenia, thereby offering a novel neuroimaging perspective for identifying white-matter functional abnormalities (Makedonov et al., 2016; Ji et al., 2019; Jiang et al., 2019).

To address this, we developed an integrative DTI and rs-fMRI framework that jointly projects white matter (WM) microstructural metrics (FA) and functional measures—skeleton-based white-matter amplitude of low-frequency fluctuations (SWALFF) and its dynamic variability (dSWALFF)—into a common space, enabling characterization of WM structure–function coupling at rest. We first applied this approach in HIV-associated ANI to identify key WM pathways exhibiting concurrent structural and functional abnormalities and to delineate patterns of coupling imbalance. We further introduced the WM dys-coupling index (WDI) to quantify structure–function deviation and examined its associations with clinical characteristics and cognitive performance. Collectively, our findings provide neuroimaging evidence supporting early identification and mechanistic interpretation of HAND, and lay a theoretical foundation for developing prognostic imaging biomarkers and precision intervention targets.

## 2 Materials and methods

### 2.1 Participants

This study was approved by the Medical Ethics Committee of Beijing You’an Hospital, Capital Medical University. All participants provided written informed consent in accordance with the Declaration of Helsinki (approval no. LL-2020-047-K). HAND diagnoses were determined according to the Frascati criteria (Antinori et al., 2007). For the identification of ANI, we applied a more conservative neurocognitive threshold suggested by Gisslén et al. (2011), defining cognitive impairment as performance ≥1.5 standard deviations below the normative mean in at least two cognitive domains. Participants classified as ANI showed no evidence of functional impairment in activities of daily living. This more stringent threshold has been proposed to improve diagnostic specificity and reduce potential overestimation of HAND prevalence. Between November 2020 and April 2024 we recruited 47 HIV-positive individuals with ANI from the outpatient clinic of the Infectious Disease Center at Beijing You’an Hospital. In addition, 48 HIV-negative healthy controls (HC) were recruited and matched to the patient group in age, sex, and years of education. Inclusion criteria for the HIV group were: (1) confirmed HIV infection, (2) Han Chinese ethnicity; (3) age 20–60 years; and (4) right-handedness. Healthy controls were required to: (1) be HIV-negative; (2) have no history of neurological or psychiatric disorders; (3) have no history of alcohol abuse or illicit drug use; and (4) show normal cognitive performance on comprehensive neuropsychological (NP) testing, with no evidence of impairment in activities of daily living. Exclusion criteria for both groups included:(1) CNS tumors, infections, cerebrovascular disease, or other systemic illnesses; (2) a history of neurological or psychiatric disorders (e.g., anxiety or depression); (3) alcohol abuse or illicit drug use; and (4) contraindications to magnetic resonance imaging (MRI). Demographic and clinical laboratory data were obtained from electronic health records (EHR), including age, sex, educational attainment, time since HIV diagnosis, duration of cART, nadir CD4^+^ T-cell count after HIV infection, plasma CD4^+^ T-cell count within 2 weeks before NP assessment, the CD4^+^/CD8^+^ ratio, and current plasma viral load.

### 2.2 Neuropsychological test

According to the Gisslén criteria, the assessment must cover at least five cognitive domains, and ANI is defined as performance at least 1.5 standard deviations below the demographically adjusted normative mean in at least two domains. Cognitive function was assessed across five domains: (1) speed of information processing (Trail-Making Test Part A, TMT-A); (2) memory, including learning and recall (Hopkins Verbal Learning Test-Revised, HVLT-R; and Brief Visuospatial Memory Test-Revised, BVMT-R); (3) attention and working memory (Continuous Performance Test-Identical Pairs, CPT-IP; Wechsler Memory Scale-III, WMS-III; and Paced Auditory Serial Addition Test, PASAT); (4) fine motor skills (Grooved Pegboard Test); and (5) verbal and language skills (Animal Naming Test). After adjusting for demographic variables (age, sex, educational level, and residential area size), corrected T-scores were derived from the raw scores of each test. Domain-specific T-scores were computed by averaging the T-scores of all tests within each domain, and a global T-score was calculated by averaging the five domain T-scores.

### 2.3 Data acquisition

All structural, functional, and diffusion tensor imaging data were acquired on a 3.0 T Siemens MRI scanner (Siemens Trio Tim, B17 software, Germany) using a dedicated 32-channel head coil. Before scanning, participants were positioned comfortably in the supine posture by adjusting the headrest and neck support, and the head was stabilized with latex pads to minimize motion during image acquisition. Prior to imaging, the researchers communicated with participants to ensure they were relaxed. Participants were instructed to keep their eyes closed while remaining awake and to wear earplugs or headphones to reduce scanner noise. T1-weighted structural images were obtained using a magnetization-prepared rapid gradient-echo (MP-RAGE) sequence with the following parameters: repetition time (TR) = 1900 ms, echo time (TE) = 2.52 ms, inversion time (TI) = 900 ms, acquisition matrix = 256 × 246, field of view (FOV) = 250 × 250 mm^2^, flip angle = 9°, voxel size = 1 × 0.9766 × 0.9766 mm^3^, and acquisition time = 4 min 18 s. rs-fMRI data were collected using a gradient-echo echo-planar imaging (GE-EPI) sequence (TR = 2000 ms, TE = 30 ms, acquisition matrix = 64 × 64, voxel size = 3.5 × 3.5 × 4.2 mm^3^, flip angle = 90°) with 35 slices and 240 time points, yielding a total scan time of 8 min 06 s. DTI data were acquired using a spin-echo echo-planar imaging (SE-EPI) sequence with TR = 9,200 ms, TE = 85 ms, slice thickness = 2 mm with no gap, 65 slices, matrix size = 112 × 112, FOV = 224 × 224 mm^2^, number of excitations = 1, spatial resolutio*n =* 2.0 × 2.0 × 2.0 mm^3^, total acquisition time = 10 min 27 s, and an anterior–posterior (AP) phase-encoding direction. Diffusion-sensitizing gradients were applied along 64 non-collinear directions with b-values of 1,000 s/mm^2^ and 0 s/mm^2^.

### 2.4 Data processing

**Figure 1:**
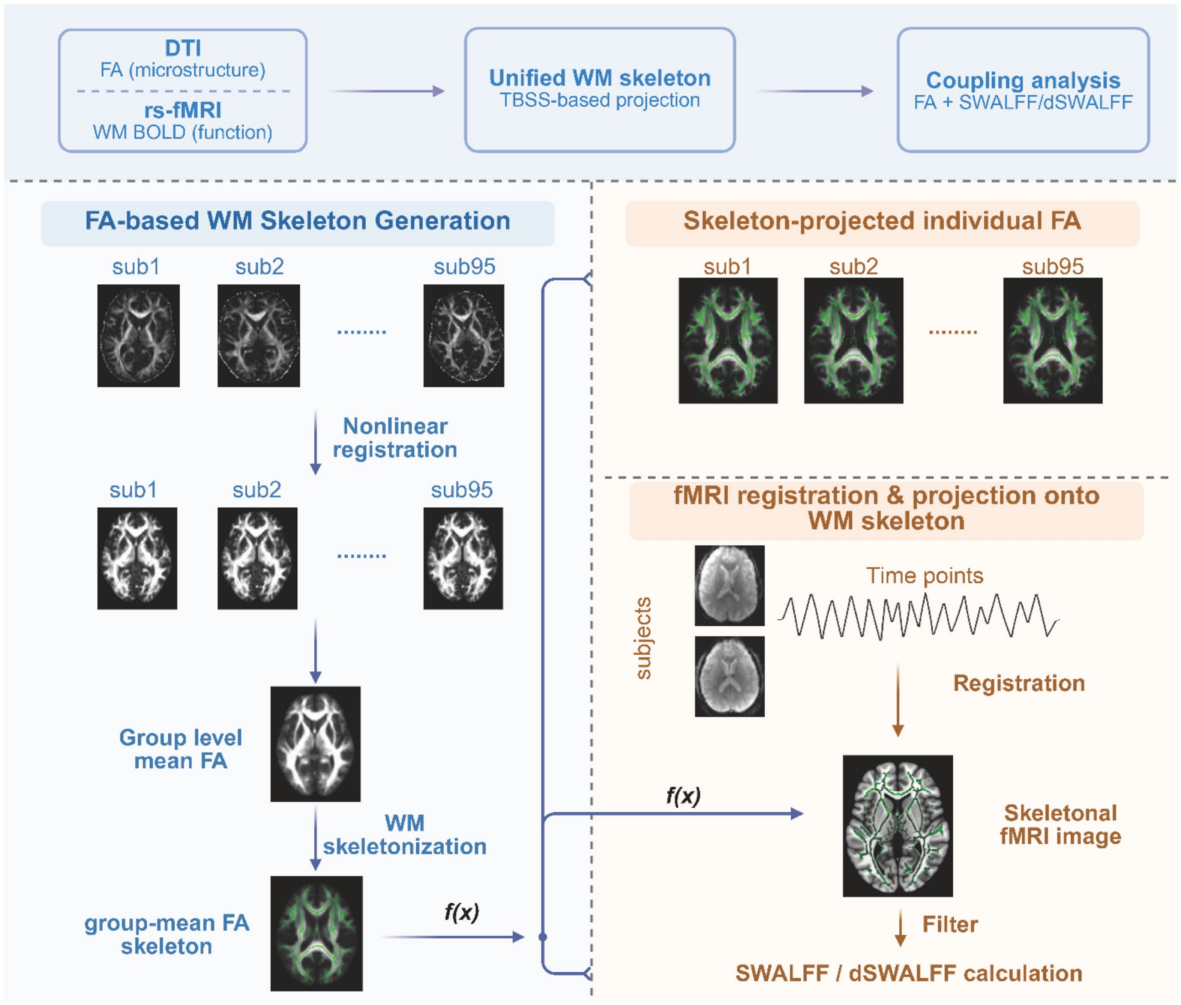
Overview of the skeleton-based DTI-fMRI fusion framework for characterizing white matter structure–function coupling. Fractional anisotropy (FA) maps derived from diffusion tensor imaging (DTI) were first nonlinearly registered to standard space, and a group-mean white matter (WM) skeleton was generated using tract-based spatial statistics (TBSS). Individual FA maps were then projected onto this skeleton to obtain skeletonized FA images. Resting-state functional magnetic resonance imaging (rs-fMRI) data underwent standard preprocessing and were registered to the corresponding FA space, after which WM BOLD signals were projected onto the same WM skeleton to achieve voxel-wise spatial alignment between structural and functional signals. Based on the skeleton-projected WM BOLD time series, skeleton-based white matter amplitude of low-frequency fluctuations (SWALFF) was calculated to quantify the strength of spontaneous low-frequency (0.01–0.08 Hz) functional activity within the WM skeleton. A sliding-window approach was further applied to compute the coefficient of variation of SWALFF across time windows, yielding dynamic SWALFF (dSWALFF) as a measure of temporal variability in WM functional activity. By jointly mapping microstructural (FA) and functional (SWALFF and dSWALFF) metrics onto a unified WM skeleton, this fusion framework enables voxel-wise assessment of white matter structure–function coupling and facilitates the identification of key pathways exhibiting coupling abnormalities in patients with HIV-associated asymptomatic neurocognitive impairment (ANI).

To achieve voxel-wise structure–function coupling within WM, we applied a DTI-fMRI fusion approach that projects DTI-derived microstructural features (FA) and WM BOLD signals from rs-fMRI onto a common WM skeleton. The workflow is illustrated in Figure 1.

#### 2.4.1 Data format conversion and quality control

Digital Imaging and Communications in Medicine (DICOM) files were converted to Neuroimaging Informatics Technology Initiative (NIfTI) format using dcm2niix, and image quality was inspected using MRIcroGL.

#### 2.4.2 DTI data processing

DTI data were first denoised and corrected for artifacts using MRtrix,^1^ including: (1) denoising; (2) Gibbs-ringing removal; (3) motion and eddy-current correction using FSL^2^ via the MRtrix command dwifslpreproc, with corresponding rotation of b-vectors according to the applied transformations; and (4) N4 bias field correction using ANTs to mitigate intensity inhomogeneity related to scanner nonuniformity. Next, microstructural metrics were estimated: (1) a brain mask was generated from the b0 image using FSL’s BET tool to constrain tensor reconstruction; and (2) diffusion tensors were fitted using MRtrix dwi2tensor, yielding four diffusion metric maps: FA, MD, axial diffusivity (AD), and RD. To address registration errors and smoothing-kernel dependence inherent to voxel-based analysis (VBA), we employed tract-based spatial statistics (TBSS), developed by the Oxford Centre for Functional MRI of the Brain (FMRIB). TBSS is a registration-based, whole-brain voxel-wise exploratory approach implemented as follows: (1) all participants’ FA images were merged into a single 4D file; (2) each FA image was linearly and non-linearly registered to standard space to obtain deformation fields; (3) a mean FA image was generated from the registered images to construct a study-specific mean FA skeleton; and (4) the mean FA skeleton was thresholded at 0.2 to create a skeleton mask, onto which each participant’s FA image was projected to obtain individual skeletonized FA maps. The same procedure was applied to AD, MD, and RD to generate the corresponding skeletonized maps.

#### 2.4.3 Rs-fMRI data processing

rs-fMRI data were preprocessed using DPARSF^3^ implemented in MATLAB 2022b (MathWorks, Natick, MA, USA) and based on Statistical Parametric Mapping (SPM12).^4^ The main steps included: (1) discarding the first 5 of 240 volumes to allow for signal equilibration; (2) slice-timing correction using interpolation to align acquisition times across slices; (3) head-motion correction by realigning all volumes to the mean functional image, with motion parameters recorded for quality control to ensure that no participant exhibited translation >3 mm or rotation >3° in any direction; (4) spatial normalization, in which the transformation from the mean functional image to the b0 image was first estimated, and the deformation field from the TBSS pipeline (individual FA to standard space) was then obtained; these transformations were sequentially applied to the motion-corrected functional images to register them to FA standard space, followed by projection onto the WM skeleton derived from the TBSS pipeline; (5) linear detrending to remove low-frequency signal drift attributable to scanner-related effects and physiological fluctuations; and (6) nuisance covariate regression using a linear model to remove cerebrospinal fluid (CSF) signals and 24 head-motion parameters (Friston-24 model).

To further reduce potential contamination from adjacent GM, the preprocessed rs-fMRI time series were projected onto the WM skeleton derived from the TBSS pipeline. This projection step restricts the analysis to the centers of major WM tracts, thereby reducing potential partial volume effects from adjacent GM and boundary voxels.

#### 2.4.4 SWALFF

To generate SWALFF maps, amplitude of low-frequency fluctuations (ALFF) was computed from the preprocessed rs-fMRI data using DPARSF. Briefly, the time series of each WM voxel was transformed from the time domain to the frequency domain to estimate spectral power; the square root of the power spectrum was then calculated to obtain the amplitude of each frequency component. ALFF for each voxel was defined as the mean amplitude within the 0.01–0.08 Hz band. To reduce inter-individual differences in global fluctuation magnitude, ALFF values were normalized by dividing the ALFF of each voxel by the mean ALFF within the WM skeleton, yielding SWALFF maps for subsequent statistical analyses.

#### 2.4.5 Dynamic SWALFF of the WM skeleton

To further quantify the temporal variability of white matter functional activity within the WM skeleton, we adopted a sliding-window approach and calculated the coefficient of variation (CV) of SWALFF across time. Selection of the window length (1/f_min) is critical to ensure that the window adequately captures low-frequency fluctuations, where f_min denotes the lowest detectable frequency in the time series and corresponds to one complete oscillatory cycle over the scan duration. Prior work indicates that window lengths shorter than 1/f_min may increase the risk of spurious fluctuations, whereas overly long windows reduce sensitivity to time-varying functional dynamics. Balancing these considerations, we selected a window length of 50 TRs (100 s) as the optimal setting. For the preprocessed rs-fMRI data, dynamic ALFF was computed in DPARSFA using a window length of 50 TRs and a step size of 5 TRs, yielding 38 windows. Within each window, SWALFF was calculated within the 0.01–0.08 Hz band. The CV of SWALFF across all windows was then computed to index temporal dynamics, resulting in dSWALFF for subsequent statistical analyses. To assess the robustness of the dSWALFF estimation, additional analyses were performed using multiple window lengths (30–50 TR) and step sizes (3–5 TR).

### 2.5 Statistical analysis

Statistical analyses of demographic/clinical characteristics and cognitive performance between the HC and ANI groups were conducted using IBM SPSS Statistics 29 (IBM Corp., Armonk, NY, USA). Categorical variables are presented as counts (percentages) and were compared using the chi-square test. Normality of continuous variables was assessed with the Shapiro–Wilk test. Normally distributed variables were presented as mean ± standard deviation (SD) and compared using the *t*-test; non-normally distributed variables were presented as median (interquartile range), median (interquartile range, IQR) and compared using the Mann–Whitney *U* test. The significance level was set at *p* < 0.05.

Group differences in WM microstructural metrics (FA, MD, AD, and RD) and WM functional measures (SWALFF and dSWALFF) were examined using a general linear model (GLM) with two-sample t-tests, controlling for age and years of education as covariates. Given the highly unbalanced sex distribution in the present cohort (all participants in the ANI group were male, and only one female was included in the HC group), sex was not included as a covariate in the statistical models to avoid unstable estimation. To further evaluate the potential impact of sex imbalance, a sensitivity analysis was performed by excluding the single female participant from the HC group and repeating all primary analyses. Statistical significance was assessed via nonparametric permutation testing (5,000 permutations), with multiple-comparison correction performed using threshold-free cluster enhancement (TFCE); results were considered significant at *p <* 0.05 [family-wise error (FWE) corrected] with a cluster size > 100 voxels. Anatomical localization and reporting of significant findings were based on the XTRACT HCP Probabilistic Tract Atlases.

To investigate the association between WM functional abnormalities and microstructural deficits, mean signal intensities were extracted from the spatially overlapping portions within each tract of the XTRACT HCP atlas between regions showing significant between-group differences in FA and those showing significant alterations in SWALFF or dSWALFF. To minimize potential bias related to spatial autocorrelation inherent to voxel-wise neuroimaging data, correlation analyses were performed using subject-level regional summary measures extracted from overlapping white-matter tracts rather than voxel-wise correlation models. Normality of FA values within the overlapping regions was assessed separately in the HC and ANI groups using the Shapiro–Wilk test. Pearson correlation was applied for normally distributed data, whereas Spearman rank correlation was used for non-normally distributed data to evaluate the association between FA and SWALFF or dSWALFF. To further ensure robustness, correlation significance was evaluated using nonparametric permutation testing (5,000 permutations), with a significance threshold of *p* < 0.05.

Building on these analyses, to further characterize abnormal WM structure–function coupling in ANI and its relationships with clinical variables and cognitive performance, we identified highly overlapping regions across the significant maps derived from the FA, SWALFF, and dSWALFF modalities along major WM tracts. These regions, which concurrently exhibited structural damage and functional alterations, were considered core loci reflecting coupling imbalance. Based on the mean signals extracted from these overlapping regions, we calculated the white matter dys-coupling index (WDI) to quantify deviations between function and structure as follows:


$$\mathrm{WDI}=\frac{\text{SWALFF}-\alpha \cdot \mathrm{FA}}{\text{SWALFF}+\alpha \cdot \mathrm{FA}}$$

Here, SWALFF reflects the intensity of WM low-frequency oscillations, FA indexes WM microstructural integrity. *α*, the tract-specific function–structure scaling coefficient, was estimated separately for each white-matter tract in the healthy control (HC) group using linear regression. Specifically, for each tract defined by the XTRACT HCP probabilistic tract atlas, mean FA and SWALFF values across HC subjects were extracted, and both variables were z-scored across subjects before regression. A linear regression model was then fitted at the group level, and the slope was defined as the tract-specific coupling coefficient (α_t_). This coefficient represents the normative structure–function coupling relationship in healthy white matter and was subsequently used to compute the WDI in ANI patients. In the WDI formula, the numerator (SWALFF - α·FA) captures the direction and magnitude of functional deviation relative to structural integrity. The denominator (SWALFF + α·FA) serves to normalize the overall activity magnitude. A WDI > 0 indicates functional hyperactivation or compensatory upregulation given a fixed structural level; a WDI ≈ 0 suggests concordance between function and structure, or their concomitant reduction; and a WDI < 0 indicates insufficient functional activity or structure–function decoupling.

To complement the XTRACT HCP atlas, recent multimodal white-matter atlases integrating microstructural, functional, and metabolic information may provide additional insights into structure–function coupling (Zhou et al., 2026). For each major WM tract (defined by the XTRACT HCP Probabilistic Tract Atlases), mean WDI values were computed to characterize tract-specific deviations in structure–function coupling. Associations between tract-specific WDI and clinical/cognitive variables were examined using Spearman rank correlation, given that the normality of these variables could not be assumed. Significance was assessed with nonparametric permutation testing (5,000 permutations) and corrected for multiple comparisons across all tested correlations using the Benjamini-Hochberg false discovery rate (FDR) procedure. Statistical significance was determined after FDR correction.

## 3 Results

### 3.1 Demographic data and cognitive performance

There were no significant differences between the HC and ANI groups in age, sex, or years of education (all *p* > 0.05). As expected, the ANI group performed significantly worse than the HC group across all cognitive domains (i.e., speed of information processing, memory [learning and recall], attention/working memory, fine motor skills, and verbal/language abilities) (all *p <* 0.05). Demographic characteristics and cognitive performance of the 95 participants are summarized in Table 1.

**Table 1:** Demographic, clinical and cognitive characteristics of the participants.

Variable	HC (*n =* 48)	ANI (*n =* 47)	*p*-value
Age (years)	30.375 ± 6.292	32.489 ± 7.380	0.136 ^a^
Male, *n* (%)	47 (97.9%)	47 (100.0%)	0.320 ^b^
Education years (years)	14.792 ± 3.274	14.638 ± 2.937	0.811 ^a^
Duration of infection (months)	N/A	53.064 ± 29.723	N/A
Duration of treatment (months)	N/A	49.957 ± 29.429	N/A
Nadir CD4^+^ count (cells/μL)	N/A	373.064 ± 201.815	N/A
CD4^+^ count (cells/μL)	N/A	565.426 ± 242.456	N/A
CD4/CD8 radio	N/A	0.680 ± 0.325	N/A
TND, n (%)	N/A	47 (100.0%)	N/A
Speed of information processing	50.771 ± 7.096	41.532 ± 9.899	< 0.001 ^a^
Memory (learning and recall)	49.797 ± 5.482	31.575 ± 7.750	< 0.001 ^a^
Attention/working memory	51.844 ± 6.306	32.053 ± 9.018	< 0.001 ^a^
Fine motor skills	51.750 ± 5.930	41.149 ± 9.818	< 0.001 ^a^
Verbal and language	56.292 ± 6.737	46.596 ± 10.147	< 0.001 ^a^

Statistical tests including: ^a^*t*-test; ^b^chi-square test. HC, healthy control; ANI, asymptomatic neurocognitive impairment; TND, virus not detectable; TIV, total intracranial volume.

### 3.2 Differences in FA, MD, AD, RD, SWALFF, and dSWALFF between ANI patients and HC

**Figure 2:**
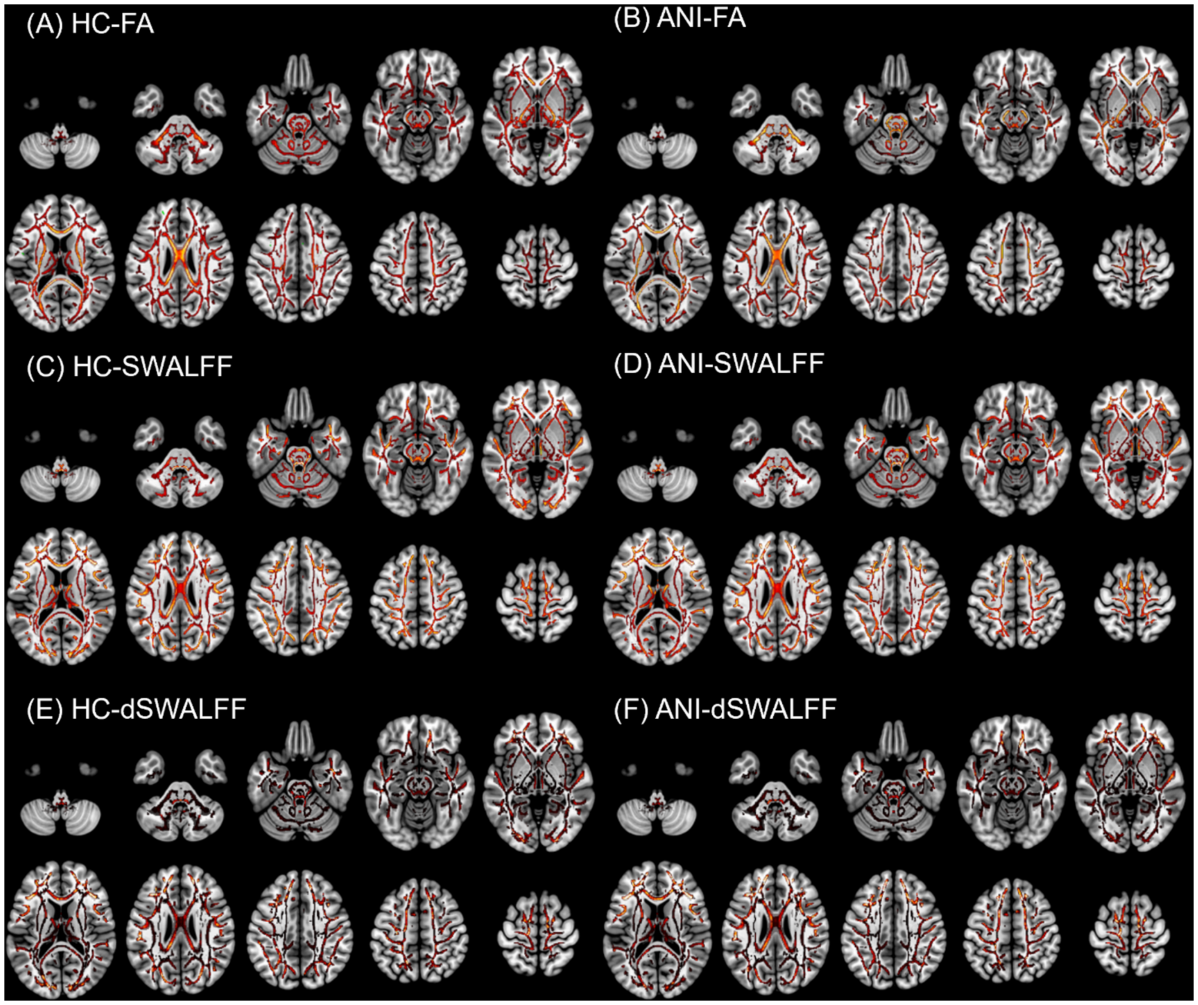
Group-average maps of FA, SWALFF, and dSWALFF in the HC and ANI groups overlaid on the white-matter skeleton. **(A)** HC-FA; **(B)** ANI-FA; **(C)** HC-SWALFF; **(D)** ANI-SWALFF; **(E)** HC-dSWALFF; **(F)** ANI-dSWALFF. All metrics are primarily distributed along the white-matter skeleton, demonstrating expected spatial patterns. These maps illustrate the overall spatial distribution of each metric, while statistical group differences are presented in subsequent figures.

**Figure 3:**
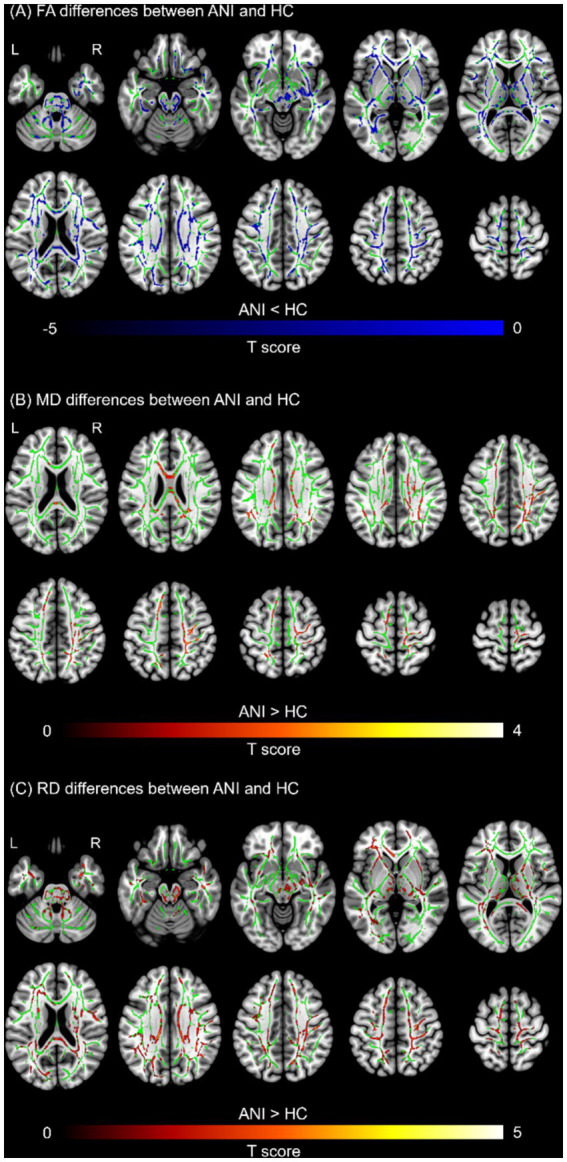
White matter microstructural differences between patients with asymptomatic neurocognitive impairment (ANI) and healthy controls (HCs). **(A)** Differences of fractional anisotropy (FA) skeleton between ANI and HCs. Two-sample *t*-tests showed widespread FA reductions (blue color) in the ANI group. These reductions were primarily located in the left inferior fronto-occipital fasciculus (IFOF), right arcuate fasciculus (AF), and left optic radiation (OR) (*P*_FWE_ < 0.05). The green color represents the mean white matter (WM) skeleton. **(B)** Differences of mean diffusivity (MD) between ANI and HCs. Significantly increased MD (red-yellow color) was observed in the ANI group, predominantly localized to the left superior longitudinal fasciculus (SLF) (*P*_FWE_ < 0.05). **(C)** Differences of radial diffusivity (RD) between ANI and HCs. Similar to MD, elevated RD (red-yellow color) was found in the left SLF in the ANI group (*P*_FWE_ < 0.05).

To facilitate interpretation of the spatial distribution of each metric, group-average maps of FA, SWALFF, and dSWALFF in both HC and ANI groups are presented in Figure 2. FWE-corrected two-sample t tests revealed widespread reductions in FA across WM in the ANI group, primarily involving the left inferior fronto-occipital fasciculus (IFOF-L; *P*_FWE_ = 0.001), right arcuate fasciculus (AF-R; *P*_FWE_ = 0.047), and left optic radiation (OR-L; *P*_FWE_ = 0.048); no WM tract exhibited a significant increase in FA (Figure 3A). To further characterize global microstructural alterations in ANI, we additionally examined MD, AD, and RD (Figures 3B,C). Compared with the HC group, the ANI group showed significantly increased MD and RD predominantly in the left superior longitudinal fasciculus (SLF-L) (MD: *P*_FWE_ = 0.021; RD: *P*_FWE_ = 0.003), with changes largely lateralized to the left hemisphere. No significant between-group differences were observed for AD. Detailed cluster characteristics, including peak MNI coordinates and sizes, are summarized in Table 2.

**Table 2:** Results in FA, MD, RD between ANI and HC.

Brain region	Peak MNI coordinate			Cluster size	*p*-value
	X	Y	Z		
FA					
Inferior Fronto-Occipital Fasciculus L	27	−41	23	51,235	0.001
Arcuate Fasciculus R	57	−37	17	192	0.047
Optic Radiation L	−17	−92	−4	128	0.048
MD					
Superior Longitudinal Fasciculus 1 L	29	−19	49	9,508	0.021
RD					
Superior Longitudinal Fasciculus 1 L	29	−42	20	43,621	0.003

Voxel-wise threshold *p* < 0.001, cluster-level family-wise error-corrected PFWE < 0.05.

**Figure 4:**
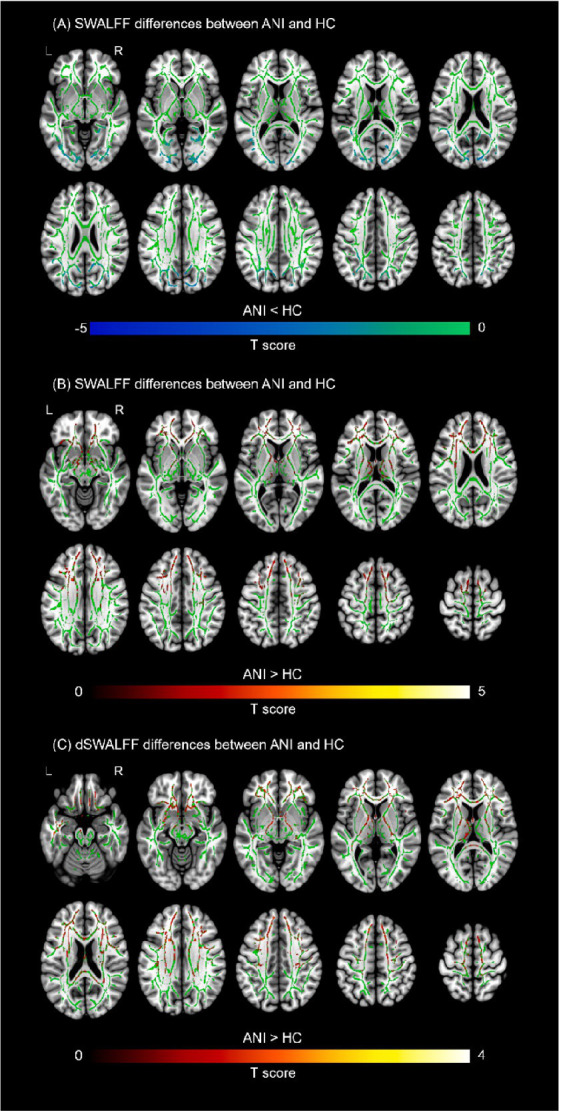
Differences in skeleton-based white matter amplitude of low-frequency fluctuation (SWALFF) and dynamic SWALFF (dSWALFF) between ANI patients and HCs. **(A)** Decreased SWALFF in the ANI group. Two-sample *t*-tests revealed significantly reduced SWALFF (blue-cyan color) in the left vertical occipital fasciculus (VOF) and forceps major (*P*_FWE_ < 0.05). The green color represents the mean white matter skeleton. **(B)** Increased SWALFF in the ANI group. Significantly increased SWALFF (red-yellow color) was observed in the forceps minor (*P*_FWE_ < 0.05). **(C)** Increased dSWALFF in the ANI group. Consistent with SWALFF alterations, dSWALFF values were significantly elevated (red-yellow color) in the forceps minor (*P*_FWE_ < 0.05).

Compared with the HC group, patients with ANI exhibited significantly reduced SWALFF in the left vertical occipital fasciculus (VOF; *P*_FWE_ < 0.001) and Forceps Major (*P*_FWE_ = 0.001; Figure 4A). Detailed cluster characteristics, including peak MNI coordinates and sizes, are summarized in Table 3.

**Table 3:** Results in SWALFF between ANI and HC.

Brain region	Peak MNI coordinate			Cluster size	*P*-value
	X	Y	Z		
ANI < HC					
Vertical Occipital Fasciculus L	−24	−84	−10	6,288	<0.001
Forceps Major	13	−93	9	5,162	0.001
ANI > HC					
Forceps Minor	−10	46	36	19,789	0.001

Voxel-wise threshold *P* < 0.001, cluster-level family-wise error-corrected PFWE < 0.05.

In contrast, increased SWALFF was observed in the forceps minor (*P*_FWE_ = 0.001; Figure 4B). Accompanied by significantly elevated dSWALFF in the same tract (*P*_FWE_ = 0.002; Figure 4C). Detailed cluster characteristics, including peak MNI coordinates and sizes, are summarized in Table 4. Similar spatial patterns were observed across different parameter settings, supporting the overall robustness of the dSWALFF findings (see Supplementary Table S1 and Supplementary Figure S1).

**Table 4:** Results in dSWALFF between ANI and HC.

Brain region	Peak MNI coordinate			Cluster size	*P*-value
	X	Y	Z		
Forceps Minor	−18	40	17	25,861	0.002

Voxel-wise threshold *P* < 0.001, cluster-level family-wise error-corrected PFWE < 0.05.

The distribution of effect sizes (Cohen’s d) for group differences in FA, SWALFF, and dSWALFF is presented in Supplementary Figure S2. Sensitivity analysis excluding the single female participant in the HC group yielded highly consistent results at the network level. Specifically, the spatial distribution and direction of group differences in RD, SWALFF, and dSWALFF remained unchanged, while FA and MD showed largely consistent patterns with only minor variations that did not affect the overall interpretation of the findings (see Supplementary Table S2).

### 3.3 Overlapping tracts of FA-altered voxels with SWALFF alterations

**Figure 5:**
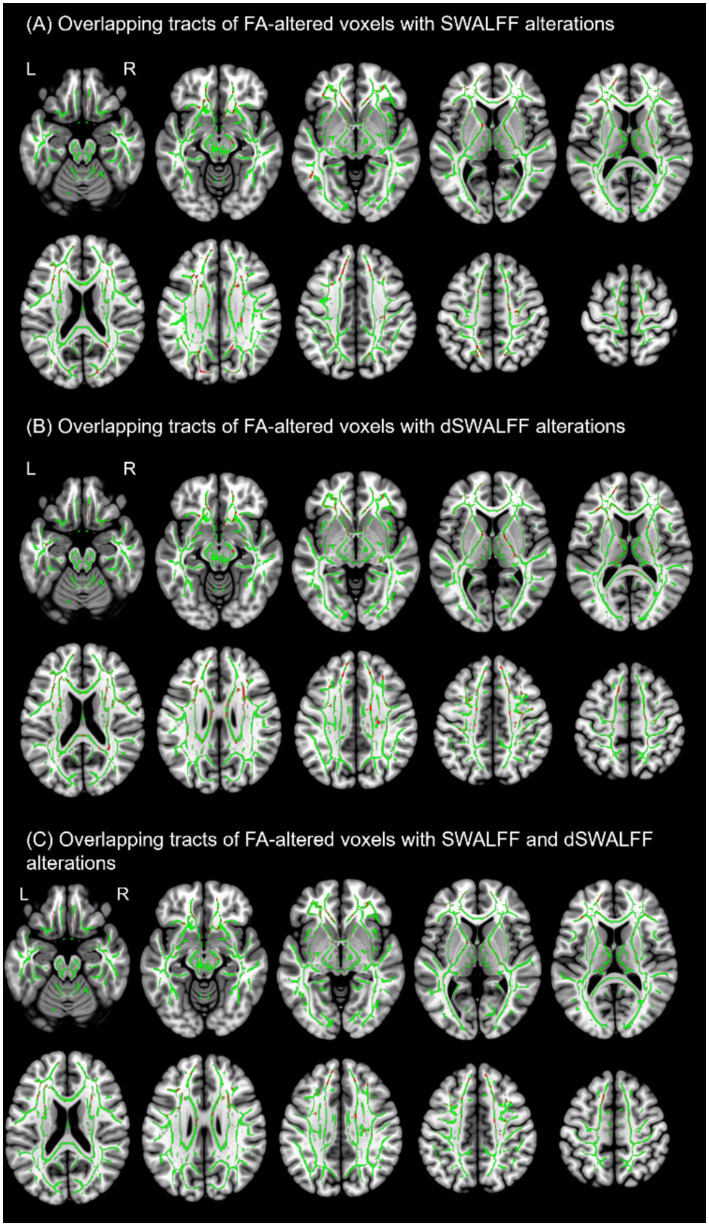
Overlapping white matter (WM) tracts exhibiting both microstructural and functional alterations in asymptomatic neurocognitive impairment (ANI). **(A)** Overlapping of FA and SWALFF alterations (red) were widely distributed across multiple white matter, with larger clusters primarily involving the left anterior thalamic radiation, forceps minor and major, and the frontal aslant tract. **(B)** Overlapping of FA and dSWALFF alterations (red) were observed, with larger clusters predominantly involving the left anterior thalamic radiation, forceps minor, and right corticospinal tract. **(C)** Triple-overlap of FA, SWALFF, and dSWALFF alterations (red), with larger clusters primarily involving the left anterior thalamic radiation, forceps minor, and left frontal aslant tract. Green represents the mean white matter skeleton.

**Figure 6:**
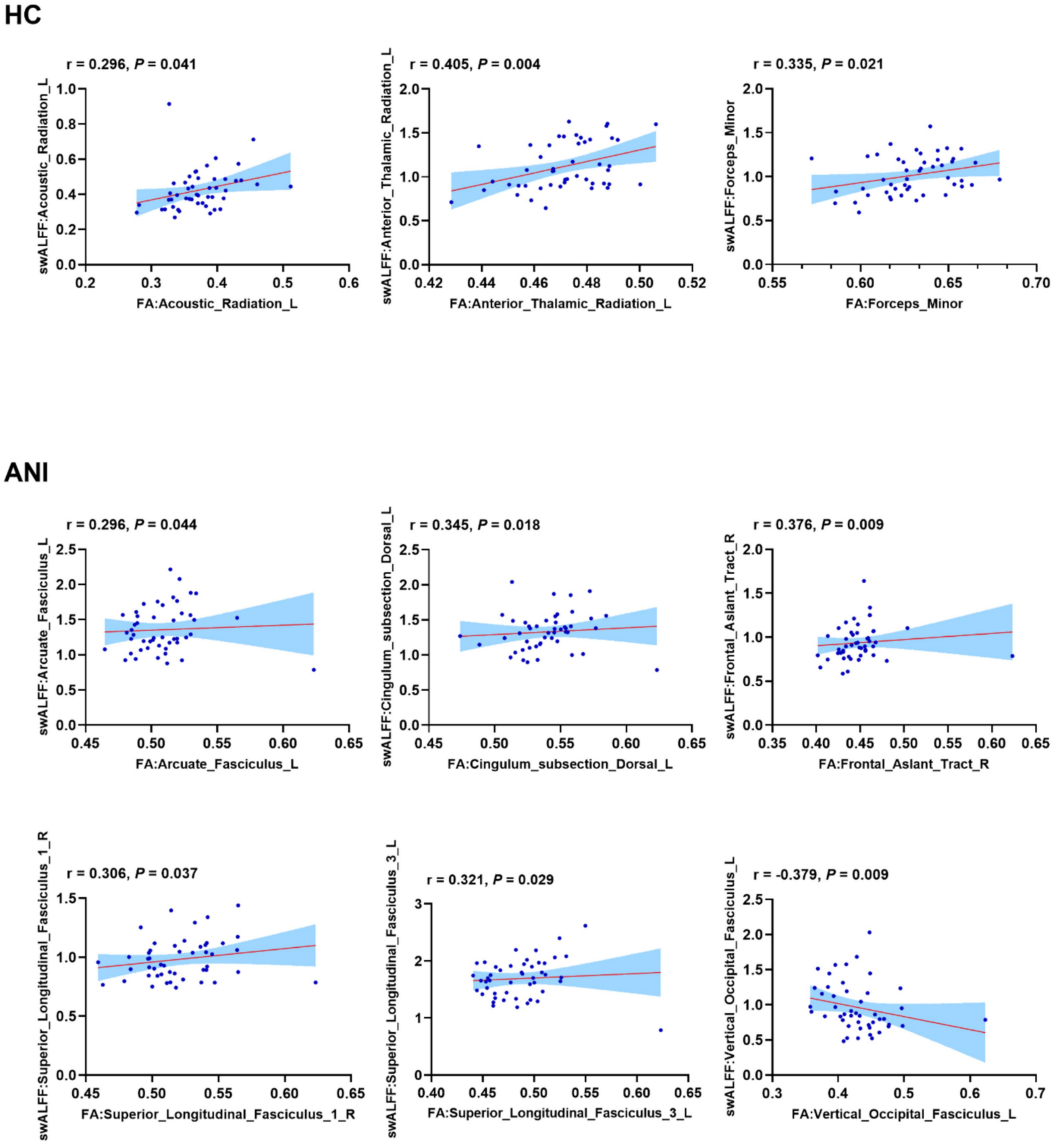
Correlations between fractional anisotropy (FA) and skeleton-based white matter amplitude of low-frequency fluctuation (SWALFF) in overlapping tracts. Scatter plots illustrating the linear relationship between FA (x-axis) and SWALFF (y-axis). In healthy controls (HC), significant positive correlations were observed in the left acoustic radiation, anterior thalamic radiation, and forceps minor. In ANI patients, positive correlations persisted in the left arcuate fasciculus, dorsal cingulum, and other tracts, while a distinct negative correlation was found in the left vertical occipital fasciculus (VOF). Statistical values (*r* and *P*) are displayed within each plot. The blue line represents the linear regression fit, and the shaded region represents the 95% confidence interval.

Figure 5A shows white matter tracts exhibiting concurrent significant alterations in FA and SWALFF, which were widely distributed, with larger overlap clusters primarily involving the left anterior thalamic radiation, forceps minor, forceps major, and the frontal aslant tract. Across all overlapping white matter regions, correlation analysis in the HC group revealed stable positive correlations between FA and SWALFF in the left acoustic radiation (r = 0.296, *p =* 0.041), anterior thalamic radiation (r = 0.405, *p =* 0.004), and forceps minor (r = 0.335, *p =* 0.021). In ANI patients, positive correlations between FA and SWALFF were maintained in the left arcuate fasciculus (r = 0.296, *p =* 0.044), left dorsal cingulum (r = 0.345, *p =* 0.018), right frontal aslant tract (r = 0.376, *p =* 0.009), and the superior longitudinal fasciculus (SLF), comprising three main branches (SLF I-III), including the left SLF I (r = 0.306, *p =* 0.037), and left SLF III (r = 0.321, *p =* 0.029). Conversely, a significant negative correlation between FA and SWALFF emerged in the left VOF (r = −0.379, *p =* 0.009), indicating a distinct structure–function coupling pattern compared to the HC group. For details, please refer to Figure 6 and Supplementary Table S3.

### 3.4 Overlapping tracts of FA-altered voxels with dSWALFF alterations

**Figure 7:**
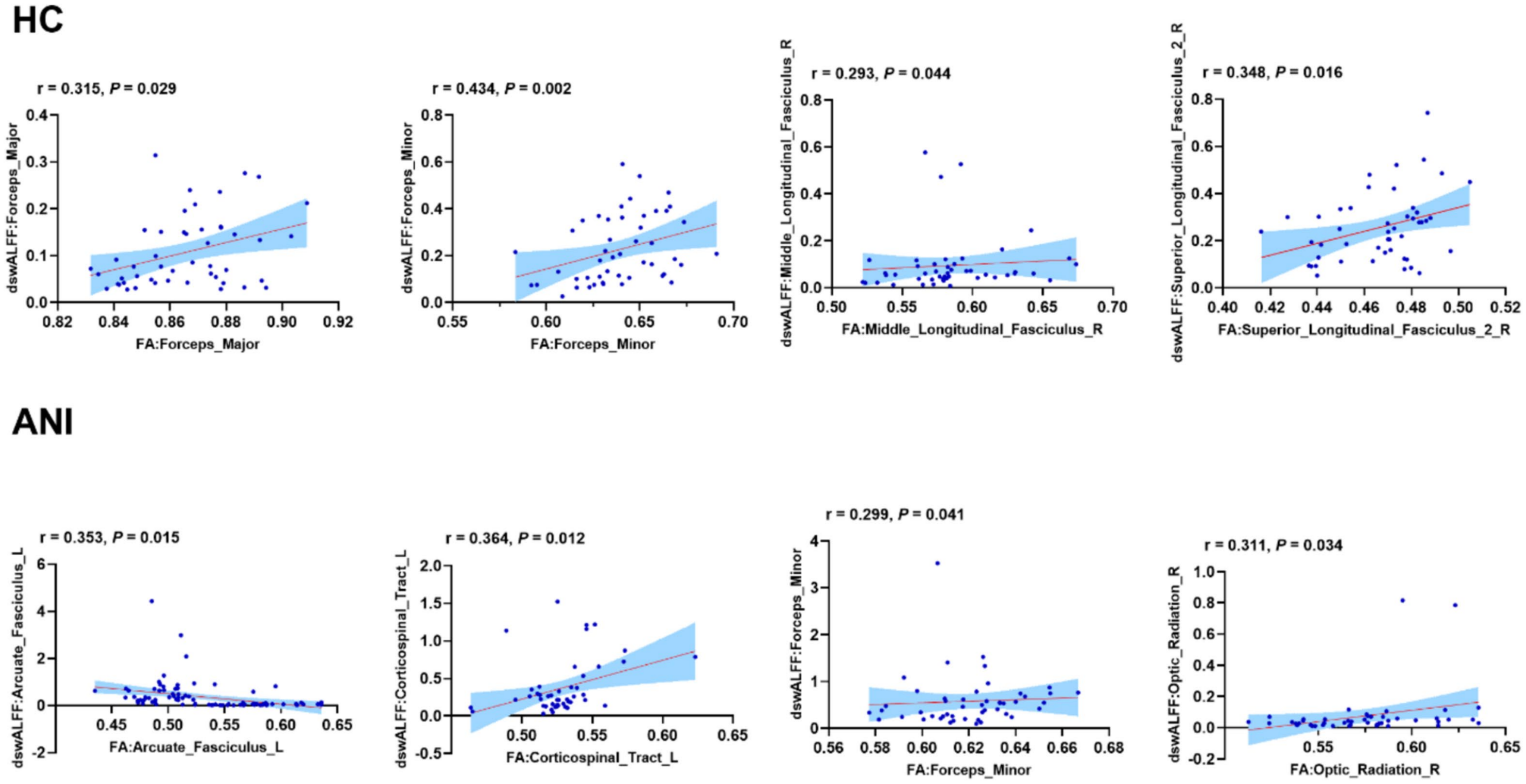
Correlations between fractional anisotropy (FA) and dynamic SWALFF (dSWALFF) in overlapping tracts. Scatter plots illustrating the associations between structural integrity (FA) and dynamic functional activity (dSWALFF). In the HC group, FA showed significant positive correlations with dSWALFF in the forceps major, forceps minor, and right superior longitudinal fasciculus (SLF). In the ANI group, significant positive correlations were observed in the left arcuate fasciculus, corticospinal tract, and optic radiation, showing a different pattern from HCs. Statistical values (*r* and *P*) are displayed within each plot. The blue line represents the linear regression fit, and the shaded region represents the 95% confidence interval.

Figure 5B shows white matter tracts exhibiting concurrent alterations in FA and dSWALFF with larger clusters predominantly involving the left anterior thalamic radiation, forceps minor, and the right corticospinal tract. Across all overlapping white matter regions, correlation analysis in the HC group revealed significant positive correlations between FA and dSWALFF in the forceps major (r = 0.315, *p =* 0.029), forceps minor (r = 0.434, *p =* 0.002), right middle longitudinal fasciculus (r = 0.293, *p =* 0.044), and right SLF II (r = 0.348, *p =* 0.016). These correlations were absent in the ANI group. Instead, ANI patients exhibited significant positive correlations between FA and dSWALFF in the left arcuate fasciculus (r = 0.353, *p =* 0.015), left corticospinal tract (r = 0.364, *p =* 0.012), forceps minor (r = 0.299, *p =* 0.041), and right optic radiation (r = 0.311, *p =* 0.034). For details, please refer to Figure 7 and Supplementary Table S4.

### 3.5 Relationship between clinical variables, cognitive performance, and WM function–structure coupling in ANI patients

**Figure 8:**
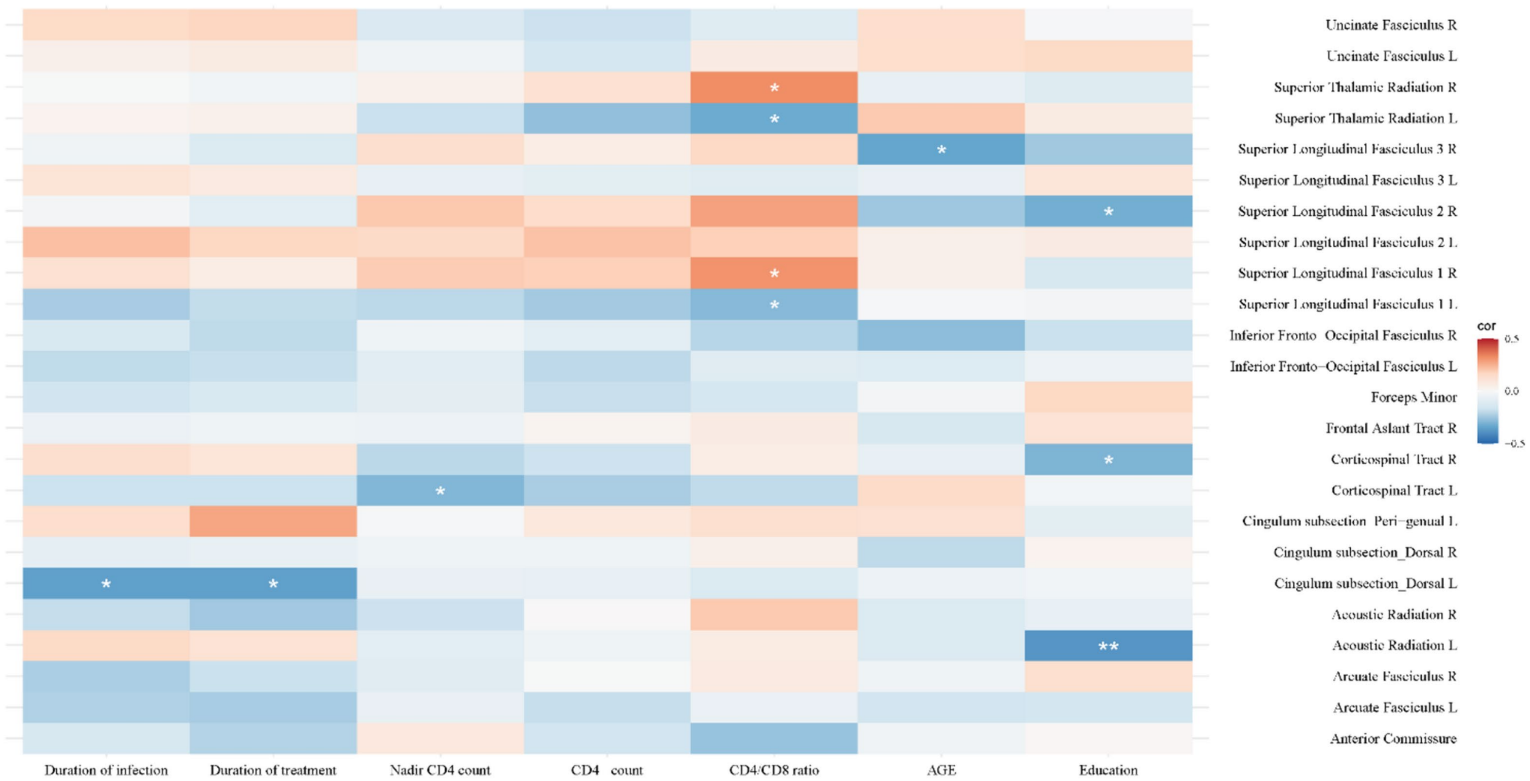
Heatmap of correlations between the White Matter Dysfunction Index (WDI) and clinical/demographic characteristics. Colors indicate correlation coefficients (red = positive, blue = negative). Significant negative correlations were observed between disease/treatment duration and WDI in the left dorsal cingulum. The CD4/CD8 ratio showed divergent patterns, with negative correlations in left-hemisphere tracts and positive correlations in right-hemisphere tracts. Additionally, education level and age exhibited significant negative associations with WDI in specific tracts such as the acoustic radiation and superior longitudinal fasciculus (SLF). ^*^P_FDR_ *<* 0.05, ^**^P_FDR_ < 0.01.

**Figure 9:**
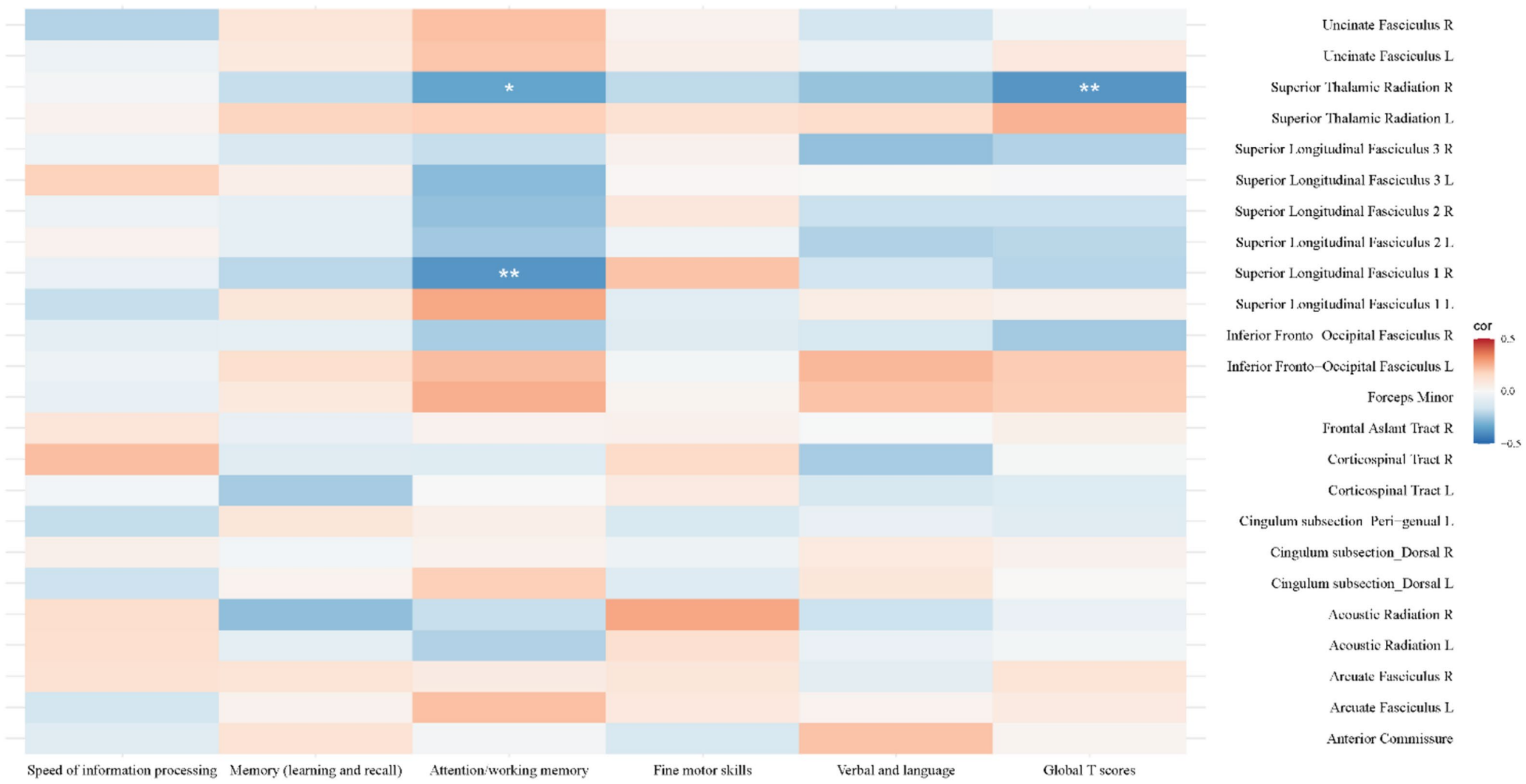
Heatmap of correlations between the White Matter Dysfunction Index (WDI) and cognitive function domains. Colors represent the correlation coefficients (red = positive, blue = negative). Significant negative correlations (blue regions) were identified between attention/working memory scores and WDI in the right superior longitudinal fasciculus (SLF) I and right superior thalamic radiation. Similarly, global T scores showed a significant negative correlation with WDI in the right superior thalamic radiation. ^*^P_FDR_ < 0.05, ^**^P_FDR_ < 0.01.

Figure 5C shows convergent FA, SWALFF, and dSWALFF alterations across white matter. Triple-overlap regions, with larger clusters predominantly involving the left anterior thalamic radiation, forceps minor, and the left frontal aslant tract, were used to extract mean FA, SWALFF, and dSWALFF values using the XTRACT HCP probabilistic tract atlas (Supplementary Table S5) for computation of the White Matter Dys-coupling Index (WDI). Following Benjamini-Hochberg false discovery rate (FDR) correction across all tested correlations, the relationships between WDI and clinical/cognitive variables were as follows: (1) Disease Duration: In the left dorsal cingulum (WDI > 0), WDI was significantly negatively correlated with both duration of infection and duration of treatment (r = −0.353, *P_FDR_ =* 0.014). (2) Immune Status: In the left corticospinal tract (WDI > 0), WDI was negatively correlated with nadir CD4 count (r = −0.294, *P_FDR_ =* 0.044). WDI in the left SLF I and left superior thalamic radiation (both WDI > 0) was negatively correlated with the CD4/CD8 ratio (r = −0.287, *P_FDR_ =* 0.049). Conversely, WDI in the right SLF I and right superior thalamic radiation (both WDI > 0) was positively correlated with the CD4/CD8 ratio (r = 0.314, *P_FDR_ =* 0.031; r = 0.326, *P_FDR_ =* 0.025). (3) Demographics: WDI values in the left acoustic radiation (WDI > 0), right corticospinal tract (WDI < 0), and right SLF II (WDI > 0) were significantly negatively correlated with years of education (r = −0.381, *P_FDR_ =* 0.008; r = −0.301, *P_FDR_ =* 0.040; r = −0.316, *P_FDR_ =* 0.030). WDI in the right SLF III (WDI > 0) was negatively correlated with age (r = −0.341, *P_FDR_ =* 0.019). (4) Cognition: WDI values in the right SLF I and right superior thalamic radiation (both WDI > 0) were significantly negatively correlated with attention/working memory scores (r = −0.378, *P_FDR_ =* 0.008; r = −0.343, *P_FDR_ =* 0.018). Additionally, WDI in the right superior thalamic radiation (WDI > 0) was negatively correlated with Global T-scores (r = −0.379, *P_FDR_ =* 0.009). For details, please refer to Figures 8, 9.

## 4 Discussion

Using a WM-skeleton-based DTI-fMRI fusion framework, this study systematically characterized voxel-wise coupling imbalance in ANI, marked by the co-occurrence of microstructural damage and functional abnormalities within WM. The ANI group exhibited widespread reductions in FA together with increases in MD and RD, suggesting diffuse microstructural injury predominantly consistent with demyelination. After projecting rs-fMRI signals onto the FA-derived WM skeleton, we further observed a bidirectional functional pattern in ANI, characterized by reduced activity within occipital afferent pathways and increased activity within prefrontal interhemispheric pathways, indicating spatially heterogeneous reorganization in the context of structural disruption. Overlapping regions of FA and SWALFF revealed a multilevel profile in which concordant decreases, compensatory upregulation, and structure–function dissociation coexisted, whereas dSWALFF highlighted instability in the dynamic regulation of WM functional activity. Moreover, the WDI quantitatively captured the magnitude of structure–function deviation and was associated with infection duration, immune status, and performance across cognitive domains, supporting its potential as an imaging biomarker for early identification of HAND with important research and clinical implications.

### 4.1 WM microstructural abnormalities: predominant demyelination in ANI

DTI-based analyses of WM microstructure indicated that individuals with HAND at the ANI stage exhibited significant decreases in FA and increases in MD and RD across multiple key WM tracts, whereas no significant changes were observed in AD, suggesting diffuse microstructural injury predominantly driven by demyelination. This pattern is consistent with prior neuroHIV findings: during the acute and early stages of HIV infection, regions such as the corpus callosum and corona radiata commonly show reduced FA accompanied by increased RD and MD, highlighting a central role of demyelination in early WM damage (Cao et al., 2015; Ragin et al., 2015; Wang et al., 2016). With longer disease duration, some studies have also reported increases in AD, implying additional axonal involvement superimposed on ongoing myelin degeneration (Leite et al., 2013; Cao et al., 2015; Kuhn et al., 2019; Chang et al., 2020; Ma et al., 2022). Collectively, our results support demyelination as a principal mechanism underlying WM pathology in ANI and provide structural evidence for the vulnerability of WM in early HAND.

### 4.2 WM functional alterations: regional hyperactivity and hypoactivity in ANI

Functional analyses showed that individuals with ANI exhibited significantly reduced SWALFF in the left VOF and the forceps major, suggesting attenuated spontaneous neural activity in posterior WM pathways dominated by occipital projections. In contrast, SWALFF and dSWALFF were significantly increased in the forceps minor, indicating enhanced functional activity within prefrontal interhemispheric pathways. This bidirectional pattern—“posterior suppression and anterior activation”—may reflect an early compensatory regulatory process.

Previous studies have suggested that the HIV-1 transactivator of transcription (Tat) protein can cross the blood–brain barrier and accumulate in the occipital cortex (Banks et al., 2005), rendering the visual network particularly vulnerable. Wang et al. reported significantly reduced resting-state functional connectivity within the visual network—especially the lateral occipital cortex—in individuals at early stages of HIV infection (Wang et al., 2011). Similarly, Ances et al. observed decreased resting cerebral blood flow (rCBF) in the visual cortex, supporting the occipital region as a highly sensitive site in early HAND (Ances et al., 2009). Moreover, magnetoencephalography and positron emission tomography (PET) studies have further demonstrated abnormal neural oscillations and chronic inflammatory responses in the occipital lobe (Vera et al., 2016; Wiesman et al., 2018). In contrast, autopsy and animal studies indicate that the prefrontal cortex in HAND is characterized by dendritic and spine degeneration, reduced synaptic density, and sustained microglial activation (Everall et al., 1991; Masliah et al., 1997; Everall et al., 1999; Li H. et al., 2023), accompanied by adaptive changes in neurotransmitter levels (Gelman et al., 2012). Neuroimaging studies confirm that prefrontal structural and functional abnormalities are closely linked to cognitive impairment in HAND (Liu et al., 2020; Sui et al., 2021; Chen et al., 2022).

In summary, the occipital attenuation and prefrontal enhancement of WM functional activity in ANI suggest region-specific plastic reorganization of neural networks, potentially reflecting early compensatory regulation alongside emerging synaptic dysfunction.

### 4.3 Multilevel patterns of white matter function–structure coupling in ANI

At the level of structure–function interactions, individuals with ANI exhibited a multilevel spectrum ranging from concordant decreases to compensatory upregulation and, ultimately, structure–function dissociation. Correlation analyses were conducted separately within white matter regions exhibiting overlapping abnormalities in FA-SWALFF and FA-dSWALFF, reflecting static and dynamic aspects of structure–function coupling, respectively. In the HC group, FA was consistently and positively correlated with both SWALFF and dSWALFF across several key WM tracts, including the acoustic radiation, anterior thalamic radiation, and forceps minor. These findings are in line with structure–function dependence models and support the role of WM microstructural integrity in sustaining functional synchrony and dynamic regulation (Honey et al., 2009; Buckner, 2010; Hagmann et al., 2010).

In contrast, ANI exhibited region-specific coupling alterations: (1) Concordant downshift—in pathways such as the arcuate fasciculus, dorsal cingulum bundle, frontal aslant tract, and superior longitudinal fasciculus, both FA and SWALFF were significantly reduced while maintaining a positive correlation comparable to that observed in HC, suggesting that although overall functional activity was attenuated, structure–function synchrony was partially preserved, consistent with a “maintenance mechanism” within the cognitive reserve framework (Barulli and Stern, 2013). (2) Compensatory upregulation: In the forceps minor and certain long-range association fibers (e.g., arcuate fasciculus, corticospinal tract, optic radiation), we observed reduced FA accompanied by elevated SWALFF or dSWALFF. The FA-SWALFF correlation diminished, while a new positive FA-dSWALFF correlation emerged. This “functional compensation in structurally compromised regions” mirrors functional reorganization in pre-manifest Huntington’s disease (McColgan et al., 2017) and may reflect dynamic transitions along the “compensation-exhaustion” continuum of white matter networks (Barulli and Stern, 2013; Vogel et al., 2023). (3) Structure–function dissociation: in the left vertical occipital fasciculus, both FA and SWALFF were reduced and their association shifted from positive to negative, indicating decoupling within occipital afferent pathways. This finding aligns with the heightened vulnerability of the visual network in early HAND (Bak et al., 2018) and may reflect reduced dependence of functional signals on the structural substrate in the presence of focal demyelination and dysfunction, leading to diminished network efficiency and impaired synchrony. Similar patterns of structure–function dissociation and reorganization have been reported across multiple neurodegenerative and psychiatric disorders (Zhang et al., 2011; McGregor and Nelson, 2019; Zou et al., 2024), further supporting spatial heterogeneity and stage-dependent remodeling of WM coupling in ANI.

Integrating static and dynamic indices (SWALFF and dSWALFF), we propose that WM networks in early HAND undergo a nonlinear trajectory from localized functional enhancement toward coupling instability: dynamic upregulation may confer short-term compensation at early stages, but progressively fails as structural injury accumulates, ultimately increasing the risk of functional exhaustion and structure–function decoupling (McGregor and Nelson, 2019; Vogel et al., 2023).

### 4.4 White matter function–structure dys-coupling: relationships with clinical and cognitive variables

By quantifying deviations in WM structure–function coupling, we proposed the WDI and further examined its clinical relevance in HAND at the ANI stage. We found that WDI was significantly associated with educational attainment, age, duration of infection and/or treatment, immune status, and cognitive performance. These findings suggest that WM coupling imbalance may be jointly shaped by disease progression and multiple clinical factors. As a quantitative measure capturing the relationship between WM functional activity and structural integrity, WDI provides additional neuroimaging evidence to support early identification of HAND.

1. Correlation between WDI and Demographics (Age, Education)

In ANI patients, WDI was significantly negatively correlated with years of education, particularly in the left acoustic radiation, right SLF II (WDI > 0), and right corticospinal tract (WDI < 0). These findings are consistent with the cognitive reserve theory, which posits that higher educational attainment enhances neural network efficiency and resilience, thereby reducing the need for compensatory functional recruitment following structural injury (Barulli and Stern, 2013). Studies have also shown that higher education is associated with a reduced risk of HAND and better cognitive performance among people living with HIV (Liu and Li, 2020; Wei et al., 2020). Additionally, WDI decreased with age, notably in the right SLF III (WDI > 0), suggesting that aging may weaken white matter compensatory capacity and destabilize structure–function coupling. This is consistent with findings of increased HAND risk in older HIV-infected individuals (Boonyagars et al., 2022; Flatt et al., 2023).

1. Correlation between WDI and Disease Duration

WDI was significantly negatively correlated with both infection duration and treatment duration, particularly in the left dorsal cingulum (WDI > 0), indicating that longer disease duration and long-term cART exposure may be associated with progressive alterations in WM structure–function coupling. Chronic HIV infection is known to cause white matter microstructural degeneration, characterized by reduced FA and elevated MD (O'Connor et al., 2017; Underwood et al., 2017). In our study, the decline in WDI may reflect a gradual weakening of functional activity relative to structural integrity, potentially indicating progressive disruption of WM functional-structural coordination. This follows a dynamic “early compensation-late exhaustion” pattern, where initial functional upregulation maintains network stability but eventually fails as structural damage accumulates, leading to reduced efficiency and functional inhibition (Zhou et al., 2025). Notably, while long-term ART aims to promote structure–function coordination, certain drugs (especially those with high CNS penetration effectiveness, CPE) may exhibit neurotoxicity, causing mitochondrial dysfunction, oxidative stress, and neuroinflammation (Yuan and Kaul, 2021). Thus, the decline in WDI over time may reflect complex changes in white matter structure–function coupling during long-term HIV infection and treatment, potentially involving both treatment-related neural stabilization and other biological processes affecting white matter networks.

1. Correlation between WDI, Immune Status, and Cognitive Performance

Regarding immune status, WDI in the left corticospinal tract (WDI > 0) was negatively correlated with nadir CD4 count, suggesting that more severe historical immune suppression is associated with greater deviations in white matter structure–function coupling (Bell et al., 2018; Yoshihara et al., 2022). Furthermore, the relationship between WDI and the CD4/CD8 ratio exhibited pathway specificity: WDI in the left SLF I and superior thalamic radiation (WDI > 0) was negatively correlated with the ratio, while right-sided pathways showed positive correlations. These findings suggest that immune status may influence deviations in white matter structure–function coupling in a pathway-dependent manner, although the underlying mechanisms require further investigation (Samboju et al., 2018). Notably, elevated WDI in the right SLF I and superior thalamic radiation was significantly associated with declines in cognitive function (attention/working memory and global cognition). This finding indicates that enhanced functional fluctuations in white matter do not necessarily reflect beneficial compensatory reorganization, but may instead represent maladaptive over-recruitment or reduced neural efficiency in the context of disrupted structure–function coupling (Bell et al., 2018; Le et al., 2023). Therefore, the functional increases observed during the ANI stage may reflect a combination of compensatory recruitment and inefficient neural processing. The functional significance of these alterations likely depends on the extent of structural injury, immune status, and the stage of disease progression.

## 5 Limitations and future directions

This study has several limitations. First, although our sample size is larger than that of many previous neuroimaging studies in HIV cohorts, it remains relatively modest for voxel-wise analyses of skeleton-based WM function and structure–function coupling. Moreover, our cohort consisted predominantly of male participants, with only one female included in the HC group. This limited sex variability precluded meaningful adjustment for sex as a covariate in the statistical models. To address this issue, we conducted a sensitivity analysis by excluding the single female participant and repeating all primary analyses. The results remained highly consistent at the network level, suggesting that the main findings are robust to potential sex-related confounding. Nevertheless, the generalizability of our findings to female populations remains to be established, and future studies with larger and more sex-balanced cohorts are warranted. Second, the cross-sectional design limits our ability to infer longitudinal trajectories or causal relationships between WM structure–function coupling alterations and cognitive impairment. Longitudinal follow-up studies are needed to clarify the role of changes in WM structure–function coupling during disease progression, including whether such changes reflect early compensatory reorganization or represent functional decoupling driven by ongoing pathology. Third, although WM functional imaging provides a promising approach for probing neural activity beyond gray matter, methodological standards for WM fMRI remain under active development. Potential contamination from adjacent GM cannot be completely excluded, although we used a skeleton-based projection strategy to mitigate partial-volume effects. Future studies should systematically evaluate alternative denoising and signal-correction strategies to further improve the robustness and reproducibility of WM functional metrics. Despite these limitations, the present framework provides a novel multimodal perspective for characterizing WM structure–function decoupling in early HAND.

## 6 Conclusion

Using a WM-skeleton-based DTI-fMRI fusion framework, we characterized voxel-wise structure–function coupling features in WM in individuals with ANI, highlighting the co-occurrence of microstructural damage and functional abnormalities. The ANI stage was characterized by widespread microstructural injury predominantly consistent with demyelination, accompanied by reduced functional activity in occipital pathways and enhanced activity in prefrontal interhemispheric pathways, forming a region-specific pattern in which compensation and decoupling coexist. We further proposed the WDI, which quantitatively captures deviations of WM functional activity relative to structural integrity and reveals a nonlinear relationship between structural degeneration and functional reorganization. WDI was significantly associated with clinical and cognitive measures, and, as an imaging marker linking functional abnormalities to underlying pathology, may have potential utility for early identification of HAND and for informing individualized interventions.

## Data Availability

The raw data supporting the conclusions of this article will be made available by the authors, without undue reservation.

## Footnotes

1
https://www.mrtrix.org/

2
https://fsl.fmrib.ox.ac.uk/fsl/fslwiki/

3
https://rfmri.org/DPARSF

4
https://www.fil.ion.ucl.ac.uk/spm/
