# Supplementary material for: Diffusion tensor imaging-functional MRI fusion reveals disrupted white matter structure–function coupling in HIV-associated asymptomatic neurocognitive impairment
Source: Front Neurosci. 2026 Apr 16;20:1793111. doi: 10.3389/fnins.2026.1793111 (PMC13128614; doi:10.3389/fnins.2026.1793111)
Supplement: Supplementary file 1 [file Data_Sheet_1.DOCX]

**Supplementary Material**

[Supplementary Figure S1. Robustness of dSWALFF findings across different sliding-window lengths 2](#_Toc225463654)

[Supplementary Figure S2. The distribution of effect sizes (Cohen's d) for group differences in FA, SWALFF, and dSWALFF 3](#_Toc225463655)

[Supplementary Table S1. Robustness analysis of dSWALFF findings across different sliding-window parameters 4](#_Toc225463656)

[Supplementary Table S2. Comparison of spatial distribution and group differences in the original cohort versus the sensitivity analysis 5](#_Toc225463657)

[Supplementary Table S3. Correlations between FA and SWALFF within overlapping white matter tracts 6](#_Toc225463658)

[Supplementary Table S4. Correlations between FA and dSWALFF within overlapping white matter tracts 7](#_Toc225463659)

[Supplementary Table S5. XTRACT HCP probabilistic tract atlas 8](#_Toc225463660)

Supplementary Figure S1. Robustness of dSWALFF findings across different sliding-window lengths


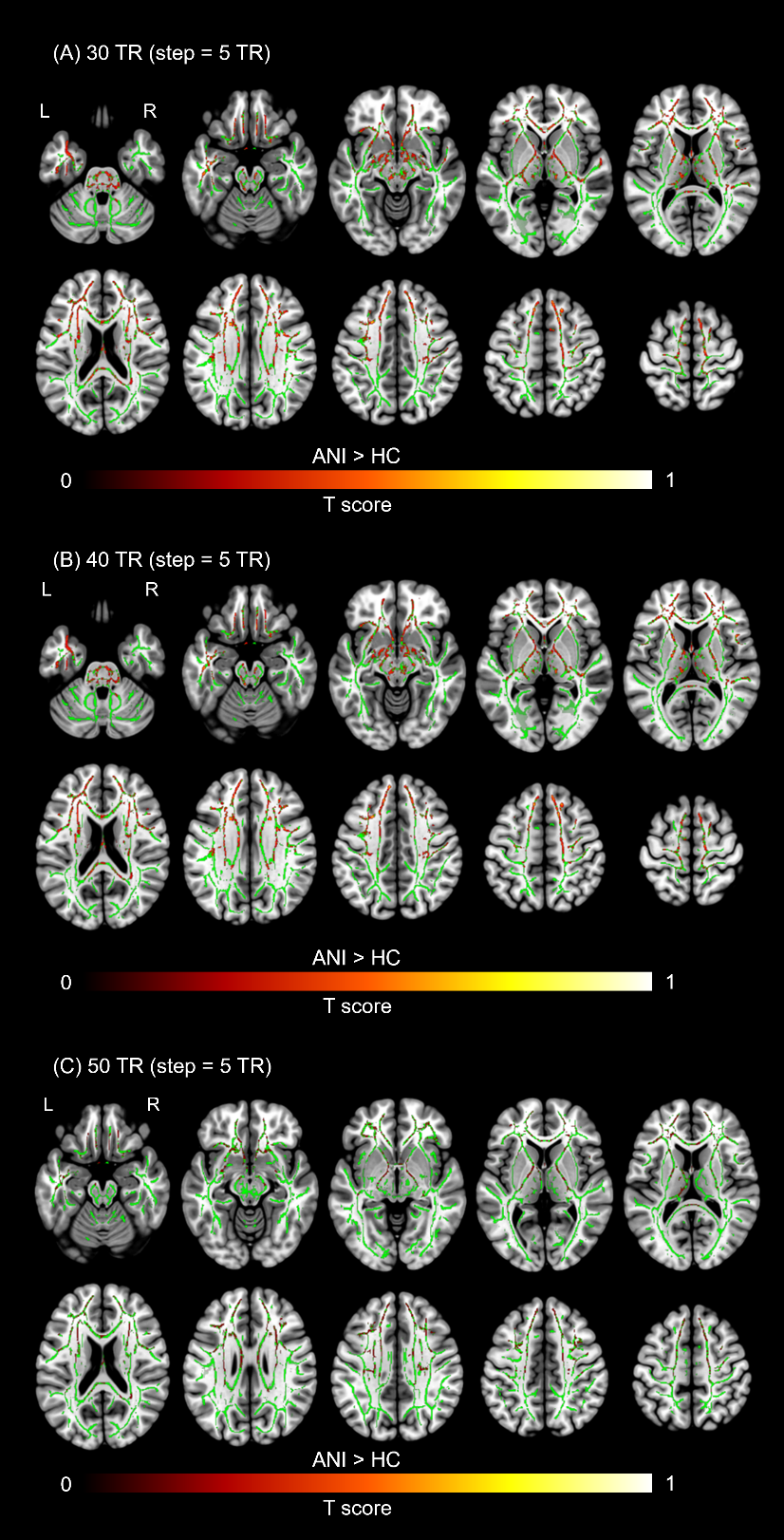


Statistical maps of dSWALFF group differences (ANI > HC) are presented for representative window lengths (30 TR, 40 TR, and 50 TR; step size = 5 TR), with 50 TR corresponding to the primary analysis. (A) 30 TR; (B) 40 TR; (C) 50 TR. Although the dominant tracts vary across window lengths, a consistent pattern of increased dSWALFF is observed across major white-matter pathways.

Supplementary Figure S2. The distribution of effect sizes (Cohen's d) for group differences in FA, SWALFF, and dSWALFF


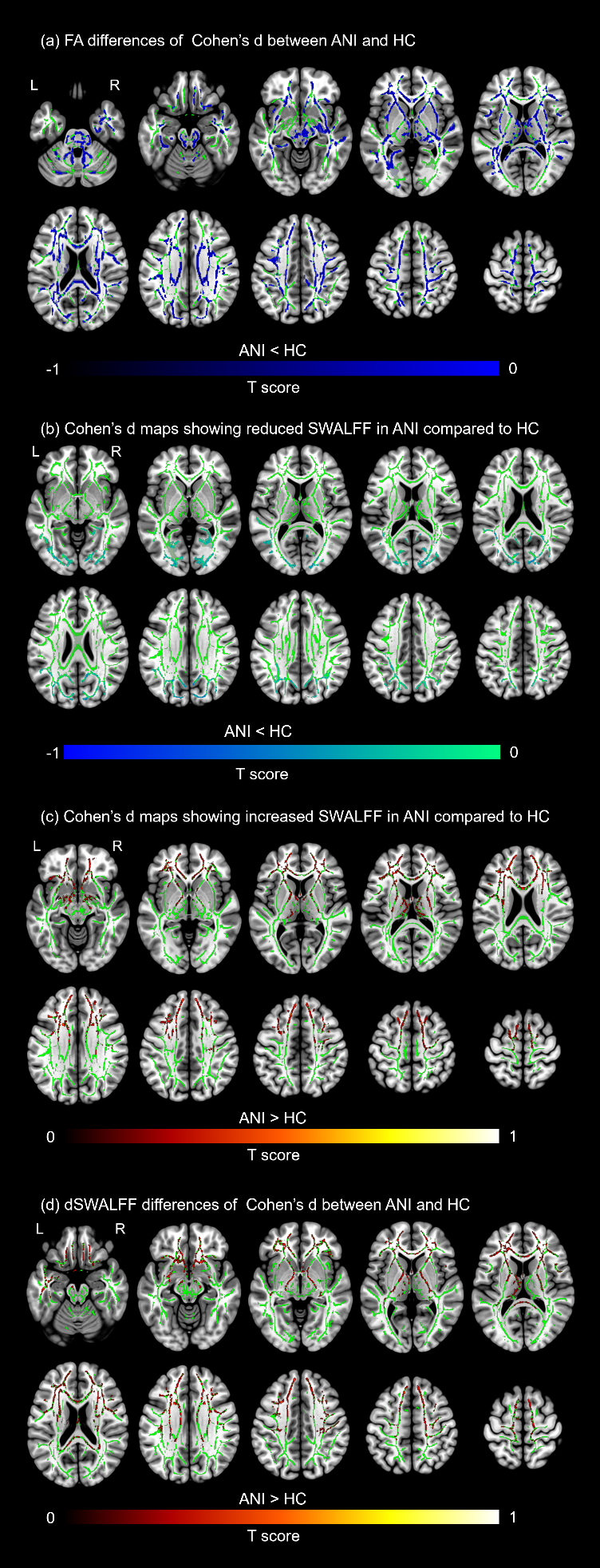


Supplementary Table S1. Robustness analysis of dSWALFF findings across different sliding-window parameters

| Window length | Step size | Significant tract(s) | Peak MNI (X, Y, Z) | Cluster size | Direction | Consistency |
| --- | --- | --- | --- | --- | --- | --- |
| 30 TR | 3 TR | Corticospinal tract (R) | (20, 49, 0) | 33026 | Increased (ANI > HC) | Largely consistent |
| 30 TR | 4 TR | Corticospinal tract (R) | (20, 49, 0) | 32913 | Increased (ANI > HC) | Largely consistent |
| 30 TR | 5 TR | Corticospinal tract (R) | (-13, 51, 25) | 33111 | Increased (ANI > HC) | Largely consistent |
| 40 TR | 3 TR | Anterior thalamic radiation (L) | (-19, 46, 14) | 31423 | Increased (ANI > HC) | Largely consistent |
| 40 TR | 4 TR | Anterior thalamic radiation (L) | (-15, 55, 9) | 31348 | Increased (ANI > HC) | Largely consistent |
| 40 TR | 5 TR | Anterior thalamic radiation (L) | (-18, 44, -6) | 31217 | Increased (ANI > HC) | Largely consistent |
| 50 TR | 5 TR | Forceps minor | (-18, 40, 17) | 25861 | Increased (ANI > HC) | Main analysis (reference) |

Note: Robustness analyses were performed using multiple sliding-window lengths (30–50 TR) and step sizes (3–5 TR). Although the exact spatial extent and peak locations varied across parameter settings, the overall pattern of increased dSWALFF in major white-matter tracts remained consistent.

Supplementary Table S2. Comparison of spatial distribution and group differences in the original cohort versus the sensitivity analysis

| **Metric** | **Main tracts (original)** | **Main tracts (sensitivity)** | **Consistency** |
| --- | --- | --- | --- |
| FA | Involvement of Widespread association and projection fibers across fronto–occipital, longitudinal, and thalamic pathways | Similar involvement of widespread association and projection fibers with minor additional clusters in frontal and limbic regions | Largely consistent |
| MD | Involvement of association and projection fibers, predominantly affecting the superior longitudinal fasciculus | Similar involvement of association and projection fibers, with a shift in dominant tracts toward cingulum and thalamic pathways | Largely consistent |
| RD | Involvement of widespread association and projection fiberss | Similar involvement of widespread association and projection fibers, with an additional small cluster in limbic pathways | Consistent |
| SWALFF | Involvement of posterior visual pathways and callosal fibers, with additional involvement of anterior interhemispheric fibers | Similar involvement of posterior visual pathways and anterior interhemispheric fibers with comparable spatial distribution | Consistent |
| dSWALFF | Involvement of anterior interhemispheric and frontal association fibers, centered on forceps minor | Similar involvement of anterior interhemispheric and frontal association fibers with comparable spatial distribution | Consistent |

Supplementary Table S3. Correlations between FA and SWALFF within overlapping white matter tracts

| **Tract** | **Group** | **FA (*x̄* ± s)** | **SWALFF (*x̄* ± s)** | **r** | ***P*** | **Correlation Type** |
| --- | --- | --- | --- | --- | --- | --- |
| Acoustic_Radiation_L | HC | 0.373 ± 0.044 | 0.425 ± 0.116 | 0.296 | 0.041 | Pearson |
| Anterior_Thalamic_Radiation_L | HC | 0.472 ± 0.016 | 1.120 ± 0.269 | 0.405 | 0.005 | Spearman |
| Forceps_Minor | HC | 0.629 ± 0.022 | 1.015 ± 0.216 | 0.335 | 0.021 | Spearman |
| Arcuate_Fasciculus_L | ANI | 0.506 ± 0.019 | 1.369 ± 0.317 | 0.296 | 0.044 | Spearman |
| Cingulum_subsection_Dorsal_L | ANI | 0.538 ± 0.022 | 1.340 ± 0.258 | 0.345 | 0.018 | Spearman |
| Frontal_Aslant_Tract_R | ANI | 0.445 ± 0.019 | 0.940 ± 0.197 | 0.376 | 0.010 | Spearman |
| Superior_Longitudinal_Fasciculus_1_R | ANI | 0.519 ± 0.026 | 0.987 ± 0.171 | 0.306 | 0.037 | Spearman |
| Superior_Longitudinal_Fasciculus_3_L | ANI | 0.484 ± 0.027 | 1.710 ± 0.316 | 0.321 | 0.029 | Spearman |
| Vertical_Occipital_Fasciculus_L | ANI | 0.425 ± 0.036 | 0.967 ± 0.345 | -0.379 | 0.009 | Spearman |

Supplementary Table S4. Correlations between FA and dSWALFF within overlapping white matter tracts

| **Tract** | **Group** | **FA (*x̄* ± s)** | **dSWALFF (*x̄* ± s)** | **r** | ***P*** | **Correlation Type** |
| --- | --- | --- | --- | --- | --- | --- |
| Forceps_Major | HC | 0.866 ± 0.019 | 0.107 ± 0.076 | 0.315 | 0.030 | Spearman |
| Forceps_Minor | HC | 0.639 ± 0.023 | 0.226 ± 0.141 | 0.434 | 0.002 | Spearman |
| Middle_Longitudinal_Fasciculus_R | HC | 0.585 ± 0.038 | 0.095 ± 0.120 | 0.293 | 0.044 | Spearman |
| Superior_Longitudinal_Fasciculus_2_R | ANI | 0.466 ± 0.020 | 0.256 ± 0.144 | 0.348 | 0.016 | Pearson |
| Arcuate_Fasciculus_L | ANI | 0.495 ± 0.023 | 0.638 ± 0.754 | 0.354 | 0.015 | Spearman |
| Corticospinal_Tract_L | ANI | 0.526 ± 0.021 | 0.362 ± 0.356 | 0.364 | 0.012 | Spearman |
| Forceps_Minor | ANI | 0.620 ± 0.021 | 0.575 ± 0.546 | 0.299 | 0.041 | Spearman |
| Optic_Radiation_R | ANI | 0.574 ± 0.030 | 0.061 ± 0.115 | 0.311 | 0.034 | Spearman |

Notes: Correlations were calculated between FA and SWALFF (or dSWALFF) within white matter tracts showing spatial overlap of significant alterations. For each tract, mean ± standard deviation (*x̄* ± s) values are reported for FA and SWALFF (or dSWALFF) within each group (HC or ANI). Normality of variables was assessed using the Shapiro–Wilk test within each group. Pearson correlation was applied when both variables were normally distributed; otherwise, Spearman rank correlation was used. Correlation significance was evaluated using nonparametric permutation testing. A significance threshold of *P* < 0.05 was applied. HC: healthy controls; ANI: asymptomatic neurocognitive impairment; FA: fractional anisotropy; SWALFF: skeleton-based white matter amplitude of low-frequency fluctuation; dSWALFF: dynamic SWALFF. Only white matter tracts showing significant spatial overlap between FA and SWALFF (or dSWALFF) alterations were included in the analysis.

Supplementary Table S5. XTRACT HCP probabilistic tract atlas

| Index | acronym | Name |
| --- | --- | --- |
| 1 | Anterior Commissure | Anterior Commissure |
| 2 | Arcuate Fasciculus L | Arcuate Fasciculus L |
| 3 | Arcuate Fasciculus R | Arcuate Fasciculus R |
| 4 | Acoustic Radiation L | Acoustic Radiation L |
| 5 | Acoustic Radiation R | Acoustic Radiation R |
| 6 | Anterior Thalamic Radiation L | Anterior Thalamic Radiation L |
| 7 | Anterior Thalamic Radiation R | Anterior Thalamic Radiation R |
| 8 | Cingulum subsection: Dorsal L | Cingulum subsection: Dorsal L |
| 9 | Cingulum subsection: Dorsal R | Cingulum subsection: Dorsal R |
| 10 | Cingulum subsection: Peri-genual L | Cingulum subsection: Peri-genual L |
| 11 | Cingulum subsection: Peri-genual R | Cingulum subsection: Peri-genual R |
| 12 | Cingulum subsection: Temporal L | Cingulum subsection: Temporal L |
| 13 | Cingulum subsection: Temporal R | Cingulum subsection: Temporal R |
| 14 | Corticospinal Tract L | Corticospinal Tract L |
| 15 | Corticospinal Tract R | Corticospinal Tract R |
| 16 | Frontal Aslant Tract L | Frontal Aslant Tract L |
| 17 | Frontal Aslant Tract R | Frontal Aslant Tract R |
| 18 | Forceps Major | Forceps Major |
| 19 | Forceps Minor | Forceps Minor |
| 20 | Fornix L | Fornix L |
| 21 | Fornix R | Fornix R |
| 22 | Inferior Fronto-Occipital Fasciculus L | Inferior Fronto-Occipital Fasciculus L |
| 23 | Inferior Fronto-Occipital Fasciculus R | Inferior Fronto-Occipital Fasciculus R |
| 24 | Inferior Longitudinal Fasciculus L | Inferior Longitudinal Fasciculus L |
| 25 | Inferior Longitudinal Fasciculus R | Inferior Longitudinal Fasciculus R |
| 26 | Middle Cerebellar Peduncle | Middle Cerebellar Peduncle |
| 27 | Middle Longitudinal Fasciculus L | Middle Longitudinal Fasciculus L |
| 28 | Middle Longitudinal Fasciculus R | Middle Longitudinal Fasciculus R |
| 29 | Optic Radiation L | Optic Radiation L |
| 30 | Optic Radiation R | Optic Radiation R |
| 31 | Superior Longitudinal Fasciculus 1 L | Superior Longitudinal Fasciculus 1 L |
| 32 | Superior Longitudinal Fasciculus 1 R | Superior Longitudinal Fasciculus 1 R |
| 33 | Superior Longitudinal Fasciculus 2 L | Superior Longitudinal Fasciculus 2 L |
| 34 | Superior Longitudinal Fasciculus 2 R | Superior Longitudinal Fasciculus 2 R |
| 35 | Superior Longitudinal Fasciculus 3 L | Superior Longitudinal Fasciculus 3 L |
| 36 | Superior Longitudinal Fasciculus 3 R | Superior Longitudinal Fasciculus 3 R |
| 37 | Superior Thalamic Radiation L | Superior Thalamic Radiation L |
| 38 | Superior Thalamic Radiation R | Superior Thalamic Radiation R |
| 39 | Uncinate Fasciculus L | Uncinate Fasciculus L |
| 40 | Uncinate Fasciculus R | Uncinate Fasciculus R |
| 41 | Vertical Occipital Fasciculus L | Vertical Occipital Fasciculus L |
| 42 | Vertical Occipital Fasciculus R | Vertical Occipital Fasciculus R |
